# Amelioration of Maternal Immune Activation-Induced Autism Relevant Behaviors by Gut Commensal *Parabacteroides goldsteinii*

**DOI:** 10.3390/ijms232113070

**Published:** 2022-10-28

**Authors:** Tzu-Lung Lin, Cha-Chen Lu, Ting-Wen Chen, Chih-Wei Huang, Jang-Jih Lu, Wei-Fan Lai, Ting-Shu Wu, Chih-Ho Lai, Hsin-Chih Lai, Ya-Lei Chen

**Affiliations:** 1 Department of Medical Biotechnology and Laboratory Science, College of Medicine, Chang Gung University, Taoyuan 33302, Taiwan; 2 Microbiota Research Center and Emerging Viral Infections Research Center, Chang Gung University, Taoyuan 33302, Taiwan; 3 Department of Chest Medicine, Internal Medicine, Fu Jen Catholic University Hospital, Fu Jen Catholic University, New Taipei City 24205, Taiwan; 4 Department of Respiratory Therapy, Fu Jen Catholic University, New Taipei City 24205, Taiwan; 5 Institute of Bioinformatics and Systems Biology, National Yang Ming Chiao Tung University, Hsinchu 300, Taiwan; 6 Department of Biological Science and Technology, National Yang Ming Chiao Tung University, Hsinchu 300, Taiwan; 7 Center For Intelligent Drug Systems and Smart Bio-Devices (IDS2B), National Yang Ming Chiao Tung University, Hsinchu 300, Taiwan; 8 Department of Laboratory Medicine and Internal Medicine, Linkou Chang Gung Memorial Hospital, Taoyuan 33305, Taiwan; 9 Department of Medicine, Chang Gung University, Taoyuan 33302, Taiwan; 10 Department of Internal Medicine, Linkou Chang Gung Memorial Hospital, Taoyuan 33305, Taiwan; 11 Department of Microbiology and Immunology, Graduate Institute of Biomedical Sciences, Chang Gung University, Taoyuan 33302, Taiwan; 12 Department of Pediatrics, Molecular Infectious Disease Research Center, Linkou Chang Gung Memorial Hospital, Taoyuan 33305, Taiwan; 13 Department of Microbiology, School of Medicine, China Medical University, Taichung 40402, Taiwan; 14 Department of Nursing, Asia University, Taichung 41354, Taiwan; 15 Research Center for Chinese Herbal Medicine and Research Center for Food and Cosmetic Safety, College of Human Ecology, Chang Gung University of Science and Technology, Taoyuan 33303, Taiwan; 16 Medical Research Center, Xiamen Chang Gung Hospital, Xiamen 361028, China; 17 Department of Biotechnology, National Kaohsiung Normal University, Kaohsiung 82446, Taiwan

**Keywords:** autism spectrum disorders, lipopolysaccharides, maternal immune activation, *Parabacteroides goldsteinii*, next-generation probiotic

## Abstract

Autism spectrum disorder (ASD) is characterized by cognitive inflexibility and social deficits. Probiotics have been demonstrated to play a promising role in managing the severity of ASD. However, there are no effective probiotics for clinical use. Identifying new probiotic strains for ameliorating ASD is therefore essential. Using the maternal immune activation (MIA)-based offspring ASD-like mouse model, a probiotic-based intervention strategy was examined in female mice. The gut commensal microbe *Parabacteroides goldsteinii* MTS01, which was previously demonstrated to exert multiple beneficial effects on chronic inflammation-related-diseases, was evaluated. Prenatal lipopolysaccharide (LPS) exposure induced leaky gut-related inflammatory phenotypes in the colon, increased LPS activity in sera, and induced autistic-like behaviors in offspring mice. By contrast, *P. goldsteinii* MTS01 treatment significantly reduced intestinal and systemic inflammation and ameliorated disease development. Transcriptomic analyses of MIA offspring indicated that in the intestine, *P. goldsteinii* MTS01 enhanced neuropeptide-related signaling and suppressed aberrant cell proliferation and inflammatory responses. In the hippocampus, *P. goldsteinii* MTS01 increased ribosomal/mitochondrial and antioxidant activities and decreased glutamate receptor signaling. Together, significant ameliorative effects of *P. goldsteinii* MTS01 on ASD relevant behaviors in MIA offspring were identified. Therefore, *P. goldsteinii* MTS01 could be developed as a next-generation probiotic for ameliorating ASD.

## 1. Introduction

Autism (autism spectrum disorder [ASD]) is a neurodevelopmental disorder characterized by social deficits, speech and nonverbal communication abnormalities, cognitive inflexibility, and repetitive/restricted sensory–motor behaviors in combination with various degrees (mild to severe) of hyperactivity, intellectual disability, and anxiety [1,2]. The reported incidence of ASD is high, occurring at an estimated rate of 1 in 88 births in the United States as of 2008 [3] and 1 in every 100 in the global population [4], indicating that the condition represents a significant medical and social problem worldwide. Despite years of studies, there are still no satisfactory medications approved for managing the core symptoms of ASD [5].

The etiology of ASD is complex, comprising multiple host genetic defects and environmental interferences characterized by maternal factors, gender differences, autoimmunity, prenatal infection, and inflammation [6,7,8,9,10]. Among these, maternal immune activation (MIA) was highlighted as an important causal factor in the later life of offspring [11,12,13]. Specifically, prenatal exposure to pro-inflammatory agents such as lipopolysaccharide (LPS) has been used as an MIA model to observe deficits in social interaction and anxiety-like behaviors in offspring [14]. Although preventive treatment with anti-inflammatory agents reduced inflammation, this did not restore structural and behavioral alterations in MIA offspring (MIAO) mice [15]. Additionally, although analyses of brain disorders including the hippocampal region of MIAO mice revealed abnormalities, the underlying molecular mechanisms were not completely characterized [16,17]. Therefore, ASD development might not be solely attributable to increased inflammatory activity, and the role of the hippocampus in the development of MIA-induced ASD in offspring also needs to be further clarified.

Recently, the gut microbiota–brain axis has been recognized as a key modulator of neuropsychiatric health, and aberrant compositional and structural shifts of microbes and their related metabolites might be causal factors of ASD [18,19,20]. Under this framework, the administration of probiotics, which are live beneficial bacteria, to reverse gut microbiota dysbiosis is expected to improve gastrointestinal dysfunction, optimize neuronal function, and consequently improve brain activities. Probiotic administration might therefore represent an effective approach to improve the abnormal behavioral profiles of children with ASD [21]. However, treatment with traditional probiotics (consortium) such as *Lactobacillus reuteri* [22], *L. acidophilus* [23], and DSF, a mixture of eight probiotic strains [24], only displayed potential ameliorative effects. Based on these findings, it is important to develop novel next-generation probiotics (NGPs) for ASD interventions as pharmaceutical applications [25,26].

In autistic subjects, a significant increase in the Firmicutes/Bacteroidetes ratio because of a reduction of the relative abundance in Bacteroidetes together with a decrease in that of *Parabacteroides* and other bacteria was reported [27,28]. In addition, the abundance of *Bacteroides* and *Parabacteroides* was also significantly decreased in germ-free mice harboring human ASD microbiomes and exhibiting ASD-like behaviors [29]. These results therefore suggested that *Bacteroides* and *Parabacteroides* species can slow the development of ASD and highlighted the importance of developing Bacteroidales-associated NGPs for ameliorating ASD-related abnormalities. In concordance, oral treatment of MIAO mice with the gut commensal bacterium *Bacteroides fragilis* corrected gut permeability, altered the microbial composition, and ameliorated ASD-related defects in communicative, stereotypic, anxiety-like, and sensorimotor behaviors [27].

The commensal bacterial strain *Parabacteroides goldsteinii* MTS01 was previously reported to ameliorate inflammation-related diseases such as obesity/metabolic syndrome and chronic obstructive pulmonary disease (COPD), and its effects were mediated through reducing LPS-related inflammation in the intestine and peripheral target organs such as adipose tissue, the liver, and the lungs [30,31,32]. In addition, *P. goldsteinii* MTS01 also restored aberrant cellular mitochondrial and ribosomal metabolic activities and maintained tissue integrity and host hemostasis [32]. Subsequent studies indicated that anti-inflammatory hypoacylated LPS derived from *P. goldsteinii* MTS01 (Pg-LPS) might act as a functional element [32]. Therefore, *P. goldsteinii* MTS01 might have the potential for development as an NGP for ameliorating LPS-induced, MIA-associated ASD relevant behaviors.

In this study, following the growth process of female MIAO mice, we longitudinally evaluated the ameliorative effects of *P. goldsteinii* MTS01 on anxiety-like behaviors and social behavioral deficits. In addition, the effects of *P. goldsteinii* MTS01 on transcriptomic patterns in both the intestine and hippocampus of MIAO mice were assessed.

## 2. Results

### 2.1. P. goldsteinii MTS01 Ameliorates Anxiety-like Behaviors

**Figure 1:**
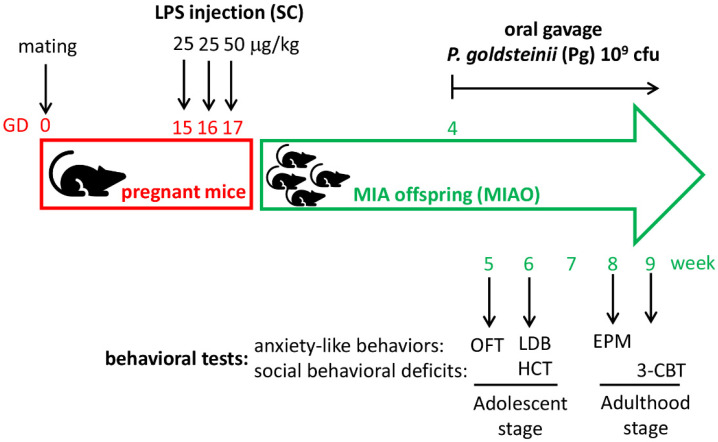
Experimental design for evaluating the ameliorative effects of *Parabacteroides goldsteinii* (Pg) MTS01 on the aberrant behaviors of offspring with lipopolysaccharide (LPS)-induced maternal immune activation (MIA). On gestation days (GD) 15, 16, and 17, pregnant mice were subcutaneously (SC) injected with 25, 25, and 50 μg/kg LPS, respectively. In the control group, pregnant mice were injected with phosphate-buffered saline. Female mice offspring were selected and grouped. Mouse offspring were orally gavaged with live Pg MTS01 at 1 × 10^9^ colony-forming units (cfu) per day for 5 days per week from week 4 through to the end of the experiment. For anxiety-like behavior assessments, the open-field test (OFT), light–dark box (LDB) test, and elevated plus maze (EPM) test were conducted at the ages of 5, 6, and 8 weeks, respectively. Regarding the social behavioral deficits evaluation, the home cage test (HCT) and three-chamber behavioral test (3-CBT) were performed at 6 and 9 weeks of age, respectively.

The experimental design on the evaluation of the effects of *P. goldsteinii* MTS01 on LPS-induced MIAO mice is presented in Figure 1. Female mice were selected and grouped as CTL group, MIAO group, and MIAO + Pg group. The abundance of *P. goldsteinii* was confirmed to be significantly increased in the MIAO + Pg group as compared to the MIAO group (Supplementary Figure S1).

**Figure 2:**
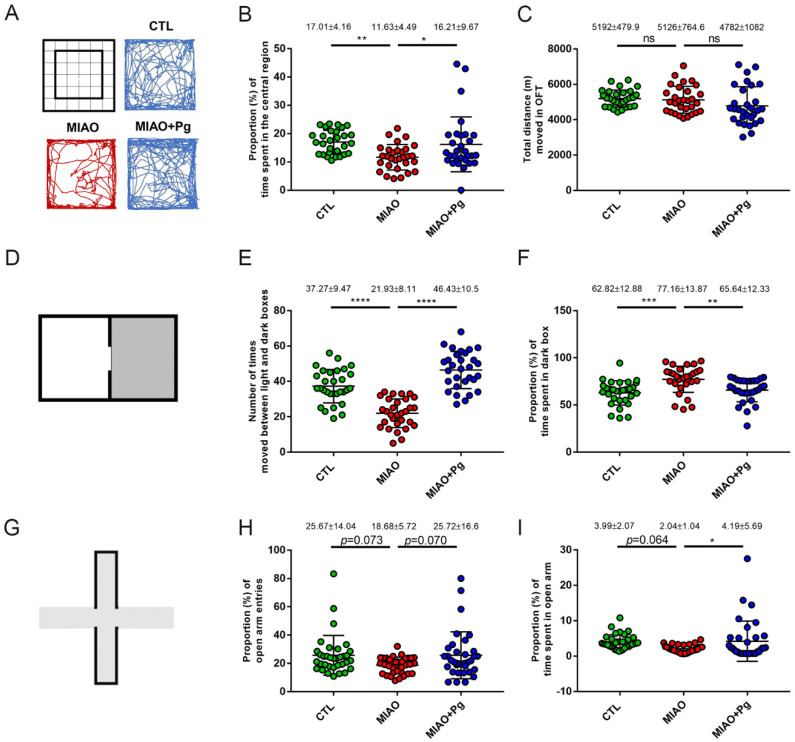
*Parabacteroides goldsteinii* (Pg) MTS01 ameliorates the anxiety-like behaviors of MIAO. Offspring from phosphate-buffered sale control group (CTL, n = 30), LPS-induced MIA group (MIAO, n = 30), and MIAO orally gavaged with Pg MTS01 (MIAO + Pg, n = 30) were evaluated for anxiety-like behaviors using the open-field test (OFT; **A**–**C**), light–dark box (**D**–**F**), and elevated plus maze (**G**–**I**). The central and peripheral regions in 36 squares as well as the representative path tracing during the OFT are presented (**A**). The proportion of time spent in the central region (**B**) and the total distances moved in the test period (**C**) are presented. A schematic representation of the LDB is presented (**D**). The number of times the mice moved between the light and dark boxes within 10 min were recorded (**E**), and the proportion of time spent in the dark box (**F**) is presented. A schematic representation of the EPM test is presented (**G**). The proportion of open arm entries (**H**) and the time spent in the open arm (**I**) are presented. Data are presented as the mean ± standard deviation. * *p* < 0.05; ** *p* < 0.01; *** *p* < 0.001; **** *p* < 0.0001; ns, not significant (one-way ANOVA, Tukey’s post hoc test).

Five weeks after birth, the OFT [33], which measures general locomotor activity levels and the willingness to explore, was performed. The results of representative path tracings are presented in Figure 2A. The proportion of time spent in the central region was 17.01% ± 4.16% in the control group. By contrast, that in the MIAO group was reduced to 11.63% ± 4.49% (*p* < 0.01; Figure 2B). Oral administration of *P. goldsteinii* MTS01 significantly increased the proportion of time spent in the central region (16.21% ± 9.67%, *p* < 0.05; Figure 2B). In comparison, no difference in locomotive activity (total distance moved in the OFT) was observed among the three groups (Figure 2C).

To evaluate whether the natural aversion of offspring mice to brightly illuminated areas and their spontaneous exploratory behavior in response to mild stress such as novel environments and light were affected by treatment, the LDB test [34] (Figure 2D) was performed at the age of 6 weeks old. As presented in Figure 2E, the time needed to move between the open and dark boxes was shorter in the MIAO group than in the control group (21.93 ± 8.11 vs. 37.27 ± 9.47, *p* < 0.0001). In comparison, *P. goldsteinii* MTS01-treated MIAO mice had a significantly higher time in moving between the boxes (46.43 ± 10.5 vs. 21.93 ± 8.11, *p* < 0.0001; Figure 2E). Specifically, the proportions of time spent in the dark box were 62.82% ± 12.88%, 77.16% ± 13.87%, and 65.64% ± 12.33% in control, MIAO, and *P. goldsteinii* MTS01-treated MIAO mice, respectively (control vs. MIAO, *p* < 0.001; MIAO vs. MIAO + *P. goldsteinii* MTS01, *p* < 0.01; Figure 2F). Together, the results of the OFT and LDB tests indicated that in adolescence, the developed anxiety-like behaviors in MIAO mice were improved by *P. goldsteinii* MTS01 treatment.

The anxiety-like behaviors of mice were further evaluated in adulthood. Eight weeks after birth, the EPM test [35] (Figure 2G), which is frequently used in neurobiological anxiety research, was performed. Although there was no statistical difference (*p* = 0.073 and 0.070, respectively), the proportion (%) of open arm entries tended to be lower in the MIAO group than in the control group, whereas *P. goldsteinii* MTS01 treatment in MIAO increased open arm entry (Figure 2H). The proportion of time spent in the open arms also tended to be lower in the MIAO group than in the control group (*p* = 0.064; Figure 2I). By contrast, oral treatment with *P. goldsteinii* MTS01 in MIAO significantly increased the time spent in the open arms (2.04% ± 1.04% vs. 4.19% ± 5.69%, *p* < 0.05; Figure 2I).

### 2.2. Social Behavioral Deficits Are Restored by P. goldsteinii MTS01

**Figure 3:**
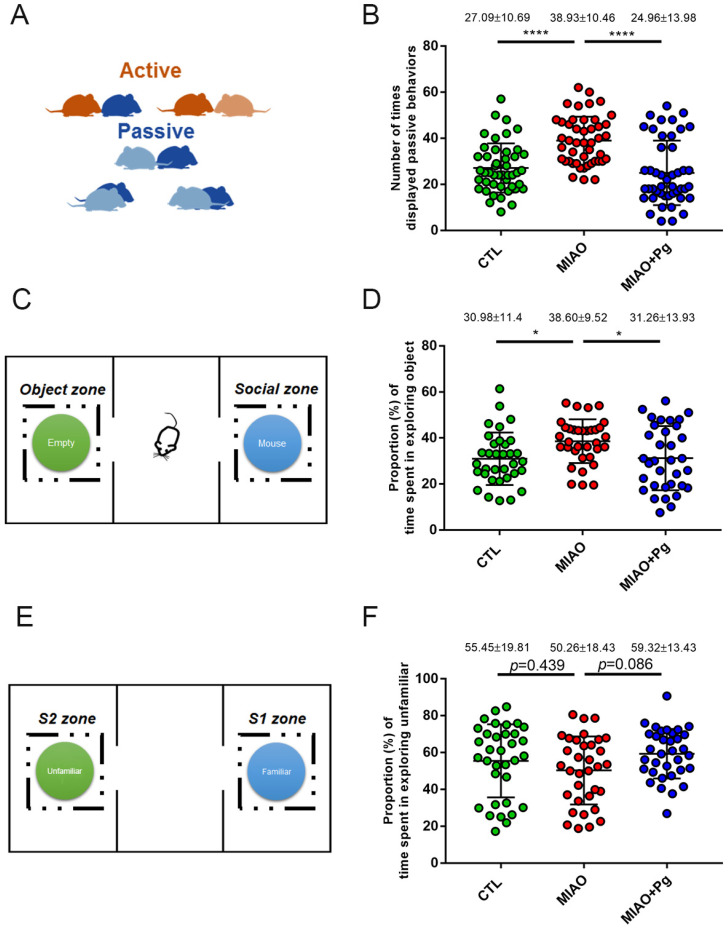
*Parabacteroides goldsteinii* (Pg) MTS01 ameliorates the social deficit behaviors of MIAO. Offspring from the phosphate-buffered saline control group (CTL), lipopolysaccharide-induced MIA group (MIAO), and MIAO orally gavaged with Pg MTS01 (MIAO + Pg) were evaluated for social deficit behaviors using the home cage test (n = 46; **A**,**B**) and three-chamber behavioral test (3-CBT, n = 34; **C**–**F**). A schematic representation of the phenotypic expression of active (nose-to-nose or nose-to-body) and passive (body-to-body) behaviors is presented (**A**). The number of times that passive behaviors were displayed was recorded (**B**). Schematic representation of the 3-CBT is presented (**C**,**E**). The proportion of time spent in the empty chamber (*Object* zone) in contrast to that in the *Social* zone was recorded (**D**). The proportion of time spent exploring unfamiliar (*S2* zone) in contrast to familiar mice (*S1* zone) is presented (**F**). Data are presented as the mean ± standard deviation. * *p* < 0.05; **** *p* < 0.0001 (one-way ANOVA, Tukey’s post hoc test).

To determine whether *P. goldsteinii* MTS01 administration also restored the social behaviors and mutual interactions of mice, MIAO mice were subjected to the HCT [36] (Figure 3A) at 6 weeks old. MIAO mice displayed more passive interactive behaviors with other mice in the home cage than with control mice (38.93 ± 10.46 times vs. 27.09 ± 10.69 times, *p* < 0.0001; Figure 3B). Oral treatment with *P. goldsteinii* MTS01 significantly reduced the number of passive behaviors (24.96 ± 13.98 times, *p* < 0.0001).

As mice grew to adulthood (9 weeks old), the behaviors of “sociability” and “social novelty” related to the propensity of mice to spend time with a previously unmet mouse compared to time spent alone in an identical but empty chamber were subsequently tested using the 3-CBT [37] (Figure 3C,E). Regarding sociability, the proportion of time spent exploring objects was significantly higher in MIAO mice than in control mice (38.60% ± 9.52% vs. 30.98% ± 11.4%, *p* < 0.05; Figure 3D). *P. goldsteinii* MTS01 administration significantly decreased the proportion of time spent exploring objects (31.26% ± 13.93%, *p* < 0.05; Figure 3D). In terms of social novelty, the proportion of time spent exploring the unfamiliar tended to be lower in MIAO mice (50.26% ± 18.43%) than in normal control offspring (55.45% ± 19.81%, *p* = 0.439; Figure 3F). The proportion of time spent exploring the unfamiliar became higher by *P. goldsteinii* MTS01 treatment (59.32% ± 13.43%), although significance was not reached (*p* = 0.086; Figure 3F). Together, the results highlighted the potential to develop *P. goldsteinii* MTS01 as an intervention for ASD.

### 2.3. P. goldsteinii MTS01 Alleviates MIA-Induced Intestinal Inflammation and Endotoxemia in Offspring

**Figure 4:**
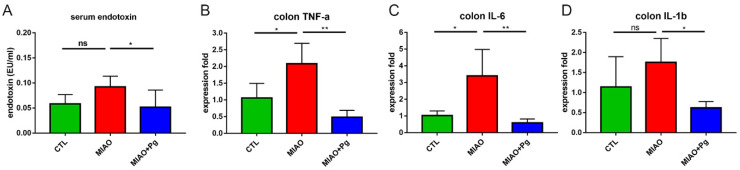
*Parabacteroides goldsteinii* (Pg) MTS01 ameliorates the gut inflammation of lipopolysaccharide (LPS)-induced MIAO. The serum endotoxin level (**A**) and colon inflammatory cytokine level (**B**–**D**) of offspring from the phosphate-buffered saline control group (CTL), LPS-induced MIA group (MIAO), and MIAO orally gavaged with Pg MTS01 (MIAO + Pg) were determined. Data are presented as the mean ± standard deviation. * *p* < 0.05; ** *p* < 0.01; ns, not significant (one-way ANOVA, Tukey’s post hoc test).

Previous studies indicated that prenatal MIA could increase the levels of interleukin-17A (IL-17A), which initiated immune-primed phenotypes in offspring that exhibited autism-like phenotypes and increased susceptibility to develop intestinal inflammation later in life [38,39]. Concordantly, intestinal inflammation was identified in MIAO mice, and *P. goldsteinii* MTS01 reduced the intestinal levels of pro-inflammatory cytokines such as IL-1β, IL-6, and TNF-α (Figure 4B–D). MIAO mice can also develop low-grade endotoxemia in sera [40]. Thus, the effects of *P. goldsteinii* MTS01 on endotoxemia in MIAO mice were evaluated. Whereas serum LPS activity was increased in MIAO mice, it was significantly reduced by *P. goldsteinii* MTS01 treatment (Figure 4A). Thus, *P. goldsteinii* MTS01 treatment reduced both local intestinal and systematic inflammation.

### 2.4. P. goldsteinii MTS01 Restores Aberrant Transcriptomes in the Intestine of MIAO Mice

The effects of *P. goldsteinii* MTS01 on the mRNA transcriptomic patterns in the intestine were next examined. To evaluate whether an a priori defined set of genes exhibits statistically significant and concordant differences among the groups, GSEA was performed. Several pathways significantly differed between the control and MIAO groups (Supplementary Figure S2A). In the MIAO group, increased immune cell responses and DNA replication, enhanced tRNA aminoacylation/ribosomal protein expression, and activated cell mitosis were identified. By contrast, sensory perception (olfactory, taste) and G-protein coupled receptor (GPCR), GABA receptor, pheromone receptor, and ion channel signaling were inhibited (Supplementary Figure S2B).

**Figure 5:**
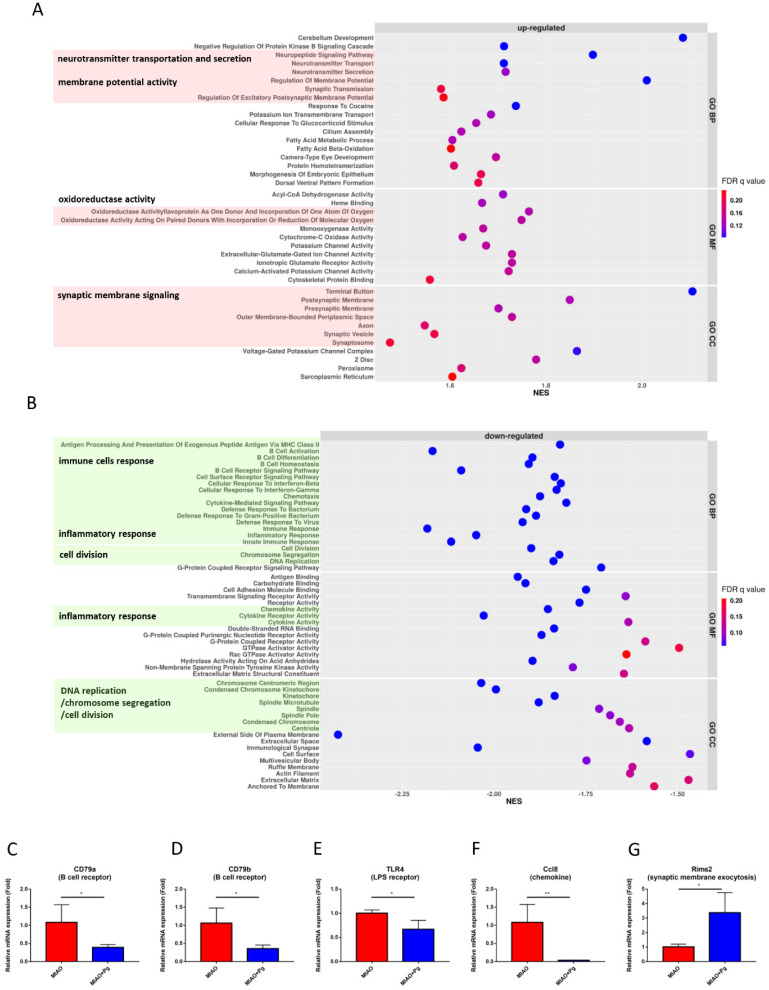
Transcriptome alterations in colon of MIAO orally gavaged with *Parabacteroides goldsteinii* MTS01. Dot plots of the significantly upregulated (normalized enrichment score [NES] > 1 and false-discovery rate [FDR] *q* value < 0.25) and downregulated ([NES] < −1 and FDR *q* value < 0.25) gene sets from gene set enrichment analysis of the MIAO + Pg group compared to the MIAO group (top 20 gene sets in GO_BP, GO_MF and GO_CC) are presented in (**A**,**B**), respectively. Gene sets with similar functions were labeled in color, and the functions were also added. Five genes contributing to the leading-edge subset within the gene sets (immune responses such as B cell receptor, lipopolysaccharide receptor, and chemokine responses; synapse responses such as synaptic membrane exocytosis) as validated by quantitative RT-PCR are presented in (**C**–**G**). Data are presented as the mean ± standard deviation. * *p* < 0.05; ** *p* < 0.01(unpaired Student’s *t*-test). MIAO + Pg, MIAO treated with *P. goldsteinii* MTS01.

*P. goldsteinii* MTS01 administration enhanced neurotransmitter transportation and secretion, membrane potential, oxidoreductase activity, and voltage-gated potassium/calcium ion channel and synaptic membrane signaling in MIAO mice (Figure 5A). In addition, *P. goldsteinii* MTS01 suppressed immune cell responses, inflammatory responses, DNA replication, chromosome segregation and cell division, and GPCR purinergic nucleotide receptor activity (Figure 5B). qPCR was used to clarify the relative expression of CD79a and CD79b (B cell receptors), TLR4 (LPS receptor), Ccl8 (chemokine), and RimS2 (synaptic membrane exocytosis). In brief, *P. goldsteinii* MTS01 reversed the aberrant transcriptomic patterns in the intestine of MIAO mice (Figure 5C–G).

### 2.5. Molecular Characterization of the Effects of P. goldsteinii MTS01 Effects in the Hippocampus of MIAO Mice

Previous studies highlighted the important role of gut commensal bacteria in the development of hippocampal neurogenesis [41,42] and identified aberrant physiological phenomena in the hippocampus in MIAO mice [43]. Whether *P. goldsteinii* MTS01 administration altered the expression of ASD-related genes in the hippocampus was analyzed. Transcriptomic analyses of hippocampus tissue-derived cells from MIAO mice with or without *P. goldsteinii* MTS01 treatment were conducted, followed by GSEA. In MIAO mice, increased expression of genes related to protein localization and targeting, positive regulation of leukocyte apoptotic processes, MHC protein complex binding, large and small ribosomal proteins, mitochondrial and respirasome protein complex components, and spliceosomal complex components were observed (Supplementary Figure S3A). By contrast, decreased expression in genes related to neuropeptide signaling pathways/endocrine system development, ion-gated channel activity, serotonin production, and GPCR and GABA receptor activity (Supplementary Figure S3B) were observed in MIAO mice in comparison to the findings in control mice.

**Figure 6:**
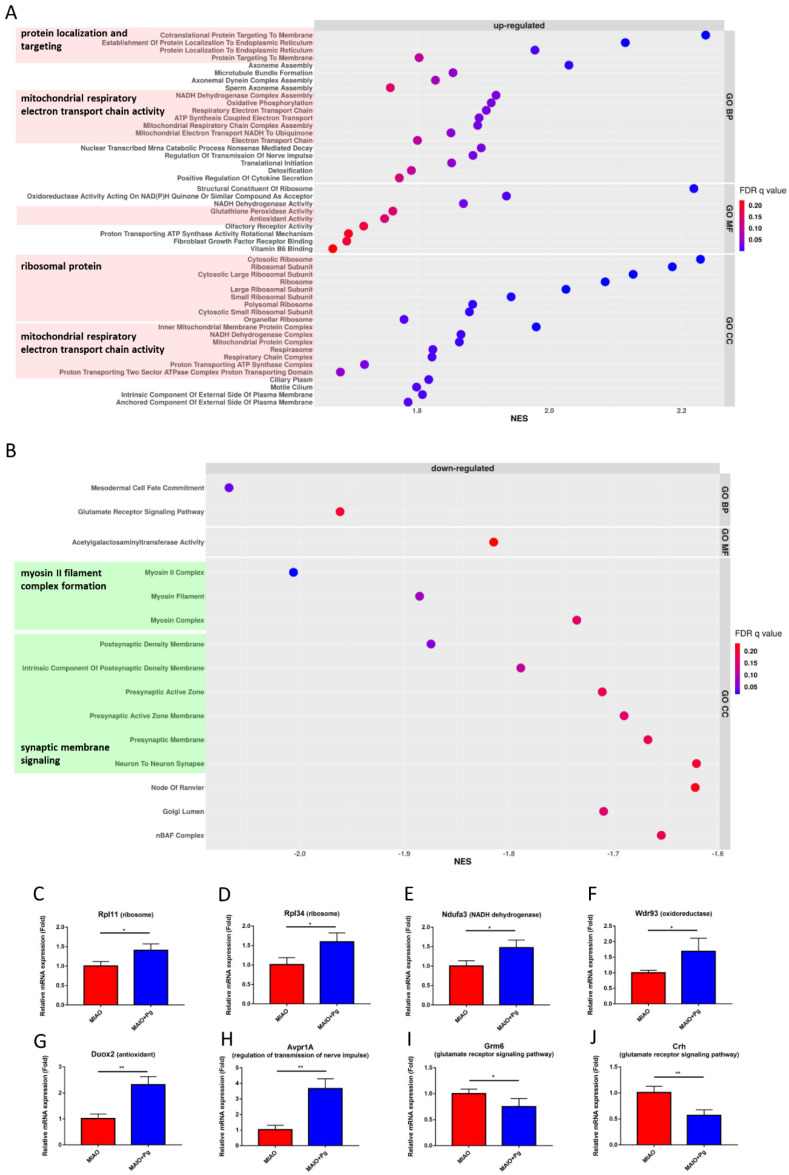
Transcriptome alterations in hippocampus of MIAO orally gavaged with *Parabacteroides goldsteinii* MTS01. Dot plots of the significantly upregulated (normalized enrichment score [NES] > 1 and false-discovery rate [FDR] *q* value < 0.25) and downregulated ([NES] < −1 and FDR *q* value < 0.25) gene sets from gene set enrichment analysis of the MIAO + Pg group compared to the MIAO group (top 20 gene sets in GO_BP, GO_MF and GO_CC) are presented in (**A**,**B**), respectively. Gene sets with similar functions were labeled in color, and the functions were also added. Eight genes contributing to the leading-edge subset within the gene sets (ribosome, oxidoreductase, NADH dehydrogenase, antioxidant, regulation of transmission of nerve impulse, and glutamate receptor signaling pathway) as validated by quantitative RT-PCR are presented in (**C**–**J**). Data are presented as the mean ± standard deviation. * *p* < 0.05; ** *p* < 0.01 (unpaired Student’s *t*-test). MIAO + Pg, MIAO treated with *P. goldsteinii* MTS01.

*P. goldsteinii* MTS01 administration in MIAO mice affected multiple pathways (Figure 6). Increased expression of genes involved in protein localization and targeting, mitochondrial respiratory electron transport chain activity and ATP synthesis, the regulation of nerve impulse transmission, detoxification, glutathione peroxidase and antioxidant/vitamin B6-binding activities, and olfactory receptor activity, in addition to genes encoding ribosomal proteins, were observed (Figure 6A). Conversely, reduced expression of genes involved in the glutamate receptor signaling pathway, myosin II filament complex formation, and synaptic membrane signaling were detected (Figure 6B).

The expression of *P. goldsteinii* MTS01-mediated genes was further validated by qPCR. Eight genes contributed to the leading-edge subset within the gene sets measured (Figure 6C–J). Of these, six genes were upregulated by *P. goldsteinii* MTS01 treatment, including Rpl34 and Rpl11 (involved in ribosome biogenesis), Wdr93 (located in mitochondrial respiratory chain complex I and involved in oxidoreductase response), Ndufa3 (related to NADH dehydrogenase activity), Duox2 (associated with antioxidant function), and Avpr1A (arginine vasopressin receptor 1A involved in regulation of transmission of nerve impulse; Figure 6C–H). In addition, the downregulation of Grm6 (Figure 6I) and Crh (Figure 6J), which are related to the glutamate receptor signaling pathway, by *P. goldsteinii* MTS01 was also validated.

### 2.6. P. goldsteinii MTS01 Modulates Immune and Neuronal Signaling in the Colon of Germ-Free Mice

As *P. goldsteinii* MTS01 was administered orally, whether *P. goldsteinii* MTS01 alone regulated the expression of genes related to neuronal development in intestinal tissue was next addressed. *P. goldsteinii* MTS01 influenced multiple pathways in germ-free mice (Supplementary Figure S4). Among these, the expression of genes related to innate immune responses and anti-microbial humoral responses were upregulated. By contrast, decreased expression of genes related to nervous system development and synaptic signaling was observed (Supplementary Figure S4). Previous results revealed a functional microbiota–neurohumoral relationship during conventionalization and suggested a delayed neuronal response that is elicited only after the microbiota accommodating homeostasis has been established [41]. In this study, the administration of *P. goldsteinii* MTS01 significantly decreased the neuronal response in the colon, implying the involvement of *P. goldsteinii* in the development of the gut–brain circuit.

## 3. Discussion

The gut microbiota was recently recognized as a key modulator of neuropsychiatric health, in which gut microbiota dysbiosis, which is closely associated with the development of gastrointestinal symptoms, might be a causal factor in altered brain/hippocampal neurogenesis and ASD development [29,44,45,46]. To ameliorate ASD, the correction of gut microbiota dysbiosis was highlighted as a treatment goal [47]. As probiotics can correct dysbiosis and improve gastrointestinal dysfunction through multiple mode of actions (MOAs), it is plausible to speculate that they can also improve the behavioral profiles of patients with ASD [24,48,49]. In addition to influencing microbiota composition, reducing gut inflammation, strengthening intestinal barrier functions, and altering mucosal immune responses [50], probiotics can also involve control of the production of metabolites, hormones, and neurotransmitters in the host [21]. However, though traditional probiotics displayed evidence of clinical efficacy against irritable bowel syndrome or ulcerative colitis, the results of previous clinical studies on ASD were inconsistent. For example, the clinical treatment of children with ASD using *L. plantarum* was reported to have a positive ameliorative effect [51]. In addition, abnormal brain plasticity and neurogenesis can be prevented by pretreatment with the Probio’Stick^®^ formulation, which modulates neuroregulatory factors and signaling pathways in the central nervous system-related stress response [52]. Furthermore, treatment with *L. reuteri* restored social deficits in several ASD mouse models by rescuing social interaction-induced synaptic plasticity via the oxytocinergic system in a vagus nerve-dependent manner [22]. Moreover, mice treated with *L. rhamnosus* JB-1 exhibited stimulation of the transcription of GABA receptors in the vagus nerve, which regulates emotional behavior [53]. Conversely, supplementation with a cocktail comprising eight probiotic strains only displayed potentially positive effects in a subset of children with ASD [24]. Furthermore, the results of a clinical meta-analysis also revealed no significant difference is patients’ anxiety scores between the probiotics and placebo groups [54].

The identification of NGPs as therapeutic agents (or more specifically “psychobiotics”) for improving the clinical treatment of aberrant neuroactivity is currently under intensive study [55]. In contrast to traditional probiotics, NGPs are beneficial bacteria basically identified from the results of recently conducted microbiota-related research. Alterations of the gut microbiota composition during ASD development, in contrast to the findings in normal controls, might reveal novel NGPs involved in the regulation of ASD pathogenesis [28]. Under strict safety regulatory control, NGPs can be used as live biotherapeutic drugs targeting psychotic diseases [26,28,49]. In one example, the administration of a non-toxigenic *Bacteroides fragilis* strain reduced bacteria-driven chronic colitis [56], protected against antibiotic-associated diarrhea [57], and also restored aberrant host metabolism and ameliorated ASD-related defects in MIAO [27]. *B. fragilis* lacking the enterotoxin gene might therefore be a good candidate for development as a potential NGP for ASD treatment [26,58].

In this study, we reported that a beneficial bacterial strain *P. goldsteinii* MTS01 reversed aberrant anxiety-like and social deficit behaviors. These changes were accompanied by reductions of intestinal and systemic (serum) inflammation, reversing intestinal abnormalities and optimizing hippocampus functions in MIAO SPF mice. MIAO mice avoided elevated areas (EPM test) and preferred darker areas over lighter areas (LDB test), indicating higher anxiety and stress in novel environments. By contrast, mice treated with *P. goldsteinii* MTS01 behaved more positively, and they tended to explore areas. In addition, MIAO mice exhibited abnormal sociability activities, i.e., reduced activity and increased passive interactions with other mice (HCT), less time spent with other rodents, and less time investigating a novel unfamiliar than a familiar one (social novelty) compared to the controls (3-CBT). By contrast, *P. goldsteinii* MTS01 treatment also significantly restored the social behavioral deficits observed in MIAO mice. Conversely, our research and previous studies revealed intestinal inflammation together with increased pro-inflammatory LPS activity in the blood of hosts with ASD [59,60]. Treatment with *P. goldsteinii* MTS01 effectively reduced this inflammation and the related abnormalities. Concordantly, similar phenomena of reducing inflammation were also observed in our previous studies on the amelioration of high-fat diet-induced obesity and metabolic syndrome [31], and smoking initiated COPD by this bacterium [32]. Although the underlying molecular mechanisms of the effects of oral *P. goldsteinii* MTS01 on inflammation were not completely clarified, Pg-LPS, an anti-inflammatory LPS derived from *P. goldsteinii* MTS01 might play an important role [32]. Pg-LPS characterized by hypoacylated lipid A moieties can alleviate the inflammation caused by pro-inflammatory *E. coli* LPS in the intestine and sera [32]. These findings might, at least partially, explain why *P. goldsteinii* MTS01 ameliorated pro-inflammatory *E. coli* LPS-induced ASD development in MIAO mice. Briefly, *P. goldsteinii* MTS01 administration reduced inflammation and ameliorated ASD-related abnormal behaviors, further highlighting its potential as an NGP for treating ASD-related diseases.

ASD is a sex-biased neurodevelopmental disorder, which is more commonly diagnosed in males than females. Differences in behavioral phenotypes between males and females were reported; therefore, their behavior should be analyzed separately. In this study, the ameliorative effect on ASD-relevant behavior of *P. goldsteinii* in female mice was prioritized to report that which was less studied in previous reports. Meanwhile, the same effect was also observed in male mice (our unpublished data) and needs further investigations.

*P. goldsteinii* MTS01 exhibited multiple MOAs in MIOA mice (Supplementary Figure S5). In addition to anti-inflammation, *P. goldsteinii* MTS01 also affected mRNA transcriptomic patterns in both the intestine and hippocampus. In the intestine of MIAO mice, inflammatory activities, cell division, and ribosomal protein expression were aberrantly increased, in addition to sensory perception, GPCR receptor signaling and ion channels were inhibited. By contrast, *P. goldsteinii* MTS01 administration reduced aberrant inflammatory activities and cell division, together with GPCR signaling, whereas it increased neurotransmitter, synaptic, oxidoreductase, and ion channel activities. In the hippocampus of MIAO mice, aberrant increases in ribosomal protein expression and mitochondrial activities were observed, whereas reduced neuropeptide signaling, ion channel activity, and GPCR and GABA/serotonin receptor activities were identified. *P. goldsteinii* MTS01 administration modulated these activities, specifically increasing ribosomal protein expression and mitochondrial, anti-oxidative/glutathione peroxidase, and olfactory receptor activities and decreasing glutamate receptor and synaptic membrane signaling. In brief, MIAO mice developed chronic inflammation-related deficits in neurotransmission and behavioral abnormalities, which were reversed by *P. goldsteinii* MTS01.

A previous study revealed beneficial effects of palmitoylethanolamide/luteolin, a combination of anti-inflammatory and antioxidant, in a mouse model of autism and in an autism case [61]. A recent study reported an anthocyanin-rich extract obtained from fruits alleviated autism-like symptoms in a mouse model of ASD and modulated the gut microbiota composition [62]. These results and our study suggested the possible linkage among antioxidant, gut microbiota, and brain.

Imbalance of the excitation and inhibition of sensory processing in the brain may lead to alterations of neural signaling, information processing, and responding behavior, which are closely associated with ASD development. Either hyper- or hyporesponsiveness in the aspect of input, cognitive, and behavioral reactivity is considered abnormal [63]. Several GPCR heteromers containing receptors sensing neurotransmitters were reported to be related to autism development. These included GABAergic, glutamatergic, dopamine, oxytocin, and 5-hydroxytryptamine receptors [5]. GPCRs are associated with different subsets of G-proteins that in turn regulate specific ion channels and trigger cAMP cascades, leading to the activation of distinctive cellular signaling pathways under differential stimulatory conditions [64]. Dysfunction in the formation and/or function of GPCR heteromers in the GPCR interactome, including olfactory receptors [65], could potentially contribute to ASD. Currently, the majority of ameliorative strategies under development mainly aim to restore the brain excitatory/inhibitory imbalance described in autism by optimizing the expression and signaling of these GPCR heteromers [5]. GPCRs might therefore represent new pharmacotherapeutic targets for autism [5].

Several neurotransmitters and specific neuropeptides are critically involved in the regulation of social behaviors. Alterations in some neurotransmitter systems that modulate brain network activities occur and potentially underlie the etiology/pathophysiology of ASD [20,66]. Among these, the imbalance between excitatory glutamatergic and inhibitory GABAergic tones has been most studied. Reduced GABA levels or the impairment of GABAergic transmission in the higher-order motor areas integrating multiple sensory modalities or aberrant increases in glutamate signaling might underlie the sensory hyperresponsiveness in patients with ASD [67,68]. Hence, GABA agonists or agents that antagonize glutamate receptors have been developed for optimizing this imbalance in the treatment of ASD [69]. Conversely, to fine-tune the excitatory/inhibitory imbalance described in autism, dopaminergic, serotonergic, oxytocinergic, and cannabinoid systems have also been addressed [5]. *P. goldsteinii* MTS01 fine-tuned many of the neurosensory systems, which may underlie the important mechanism of amelioration. In addition, although the gut microbiota is involved in the production of neuroactive compounds such as GABA and glutamate [20], whether *P. goldsteinii* MTS01 produces some of these neurotransmitters remains to be further studied.

Multiple other abnormal physiological activities are involved in ASD development. Increased enterocyte and Paneth cell counts in the intestinal epithelium and lamina propia, together with increased crypt cell proliferation, have been found in autistic children [70]. In parallel, increased brain growth and mutations in autism were also reported [71]. Meanwhile, a growing body of evidence from whole-genome and whole-exome sequencing has suggested a linkage of several ASD susceptibility genes for potassium channels in subjects with ASD. Genetic dysfunction of potassium channels may be involved in the altered neuronal excitability and abnormal brain function in the pathogenesis of ASD [72,73]. In addition, the roles of ribosomal and mitochondria-related activities were also highlighted. Abnormal (either increased or decreased) ribosomal protein gene expression and RNA spliceosome activity in the brain [72,74], together with aberrant mitochondrion-related electron transportation chain and oxidative phosphorylation/respiratory function activities [75], was reported to be closely associated with the development of neurodevelopmental pathologies in hosts with ASD [76]. In this study, GSEA revealed the upregulation of genes involved in ribosomal biogenesis and mitochondrial activities in MIAO mice, and their expression was further enhanced by *P. goldsteinii* MTS01. Such phenomena warrant further detailed studies. Another important factor involved in ASD is the neuronal activities responsible for regulating localized protein synthesis within dendrites and the post-translational modification of synaptic molecules. These were associated with regulating synaptic function and allowing neuronal circuits to respond dynamically to stimuli [66]. Dysregulation of such signaling pathways was ameliorated by *P. goldsteinii* MTS01, which may also play a key role in ameliorating ASD. *P. goldsteinii* MTS01 optimally delayed the expression of neuron development-related genes in the intestine of germ-free mice, indicating the important effects of *P. goldsteinii* MTS01 on the homeostatic development of neuroma system in the intestine.

Taken together, *P. goldsteinii* MTS01 administration appeared to optimize multiple activities in both the intestine and hippocampus of MIAO mice, simultaneously leading to the maintenance of cellular homeostasis, optimal turnover of biochemical macromolecules, increased cellular antioxidant activities, and optimization of the signaling pathways related to the control of neurobehaviors. Therefore, *P. goldsteinii* MTS01 has the potential for development as an NGP or live biotherapeutic product for the potential prevention or treatment of ASD. In addition, the active component(s) of *P. goldsteinii* MTS01, as well as the underlying molecular mechanisms that control multiple regulatory activities and restore aberrant functions and phenotypes in ASD models, remain to be further characterized.

## 4. Materials and Methods

### 4.1. Animals

The animal experiments performed in this study were approved by the Institutional Review Board of National Kaohsiung Normal University, Taiwan and performed according to the instructions of the Animal Protection Act (Kaohsiung, Taiwan). In the study 8- and 10-week-old female C57BL/6J mice were purchased from the National Laboratory Animal Center (NLAC, Taipei, Taiwan) and bred under specific pathogen-free (SPF) conditions with a strict 12-h light (8–19 o’clock)/dark (20–7 o’clock) cycle (22–24 °C; 55–65% humidity). The experiments were performed from 10:00 AM to 12:00 PM. All mice were individually acclimated at cage for 3–5 days. For mating, the female acclimated at another cage that harbored the odor of male mouse for 3 days followed by 2 female mice being mated with 1 male mouse. On the day that vaginal plugs appeared, females were housed separately, and this day was designated as gestational day 0 (GD0). The offspring were routinely housed in separate cages with 2–3 females per cage and fed a standard food diet (Oriental Yeast Co., Chiba, Japan). Germ-free C57BL/6JNarl mice were purchased from NLAC. Mice were maintained in a vinyl isolator and confirmed their germ-free status by culturing feces, bedding, and drinking water in thioglycollate medium (DIFCO, Camarillo, CA, USA).

### 4.2. Experimental Design

*Escherichia coli* LPS is commonly used for inducing maternal immune activation of C57BL/6J mice at prenatal infection [11]. To evaluate the appropriate doses, *E. coli* O55:B5 LPS ranging from 25 to 80 μg /kg were tested before formal experiments. Eventually, pregnant mice injecting subcutaneously (sc) *E. coli* O55:B5 LPS three times (GD15, 25 μg/kg; GD16, 25 μg/kg; and GD17, 50 μg/kg; Figure 1), with optimal offspring survival rate >92% and maternal abortion rate <10%, were used for MIA induction in this study.

Female MIAO mice were selected and grouped, followed by behavioral experiments. Offspring mice derived from pregnant mice subcutaneously injected with phosphate-buffered saline were used as the normal control. For MIAO mice treated with *P. goldsteinii* MTS01, each offspring was orally gavaged with 1 × 10^9^ colony-forming units (cfu) of live bacteria once per day for five days per week (from week 4 to the end of the experiment). Control and MIAO that served as control were gavaged with PBS.

Germ-free mice were orally administrated with *P. goldsteinii* MTS01 (5 × 10^8^ cfu) once at 8 weeks old. After 2 weeks of mono-colonization, mice were sacrificed, and their colons were collected.

### 4.3. P. goldsteinii Cultivation

*P. goldsteinii* MTS01 was isolated from the feces of a healthy adult who received *P. goldsteinii* MTS01 isolated from a healthy mouse [32] for 6 months. Bacteria were grown at 37 °C in a Whitley DG250 anaerobic chamber (Don Whitley, Bingley, UK) with mixed anaerobic gas (5% carbon dioxide, 5% hydrogen, 90% nitrogen). The anaerobic condition was confirmed using an anaerobic indicator (Oxoid, Basingstoke, UK). *P. goldsteinii* MTS01 was cultivated on anaerobic blood agar (Creative, New Taipei City, Taiwan) and liquid thioglycollate medium (BD, Franklin Lakes, NJ, USA).

### 4.4. Behavioral Tests

In this study, a total of 17 (MIAO), 18 (MIAO + Pg), and 13 (CTL) littermates were used for behavioral tests (details in Supplementary Table S1). The mice from different littermates were maintained at body weights (ranging from 16 to 20 g, aged 5 to 8 weeks old), locomotor activities (ranging from 49 to 52 m observed by OFT at 5 weeks of age), and muscle strength (scores ≥ 4 points, measured by standard wire-hanging evaluation at 8 weeks of age). All tested mice had similar physiological conditions throughout the study.

Individually housed female offspring completed the open-field test (OFT), light–dark box (LDB) test, and home cage test (HCT) during adolescence (5 weeks old) as well as the three-chamber behavioral test (3-CBT) and elevated plus maze (EPM) test in adulthood (8 weeks old) [77]. Each behavioral experiment was separated by 2 days. All tests were automatically video-recorded (iCATCH, DVR-413DH-J; Panasonic, Taipei, Taiwan) and imaged using software (iWATCH DVR, v2; Panasonic) per standard protocols [77]. Before testing, individual mice were allowed to move freely for 10 min at individual equipment. Briefly, the OFT was performed at 60 × 60 cm of testing area including 36 squares (10 × 10 cm per square; outer, 20 squares; inner, 16 squares) illuminated at 65 lux for 10 min. For LDB, the light–dark box (45 × 27 × 454 cm) was set to 45 and <1 lux, respectively. The mice faced the open door (7.5 × 7.5 cm) at the light area to start the test for 10 min. The EPM apparatus was set to open arms (30 × 5 cm, 100 lux, without wall) and closed arms (30 × 5 cm, 90 lux), surrounded by 16-cm high whitewalls, no shadow) at 50 cm above the floor. The central platform, which was located at a space between the open and closed arms, was a 5 × 5 cm square with illumination of 100 lux. The test was started with mice facing the open arm and performed for 10 min. For HCT, the interactions of 2 tested mice (sibling) were recorded every 30 s for 30 min. Totally, 122 measurements were recorded. For the 3-CBT, the mice were placed at one of the wire-mesh cages (20 × 20 × 45 cm, every cage) illuminated by 70 lux at the start. Another empty cage recognized as an object or the mouse from a different home cage with a similar body weight, age, and sex, was designated as unfamiliar.

### 4.5. Endotoxin Detection

Serum LPS levels were measured using a murine *HEK*-*Blue*™ LPS Detection *Kit* (InvivoGen, San Diego, CA, USA) based on the manufacturer’s instructions.

### 4.6. Quantification of Gene Expression Levels and Abundance of P. goldsteinii

At sacrifice, mice were anesthetized with isoflurane and blood was collected via intracardiac puncture. The colon and hippocampus tissues were collected in RNAlater solution (Sigma-Aldrich, St. Louis, MO, USA). Total RNA was extracted using a Genezol TriRNA pure kit (Geneaid, New Taipei City, Taiwan) per manufacturer’s protocol. RNA was reverse-transcribed into cDNA using a Quant II fast reverse transcriptase kit (BioTools, New Taipei City, Taiwan). The resulting cDNA was used as a template for quantitative PCR (qPCR) using the primers listed in Supplementary Table S2. The PCR conditions were as follows: initial pre-incubation-step at 95 °C for 3 min; 50 cycles at 95 °C for 10 s, 60 °C for 20 s, and 72 °C for 5 s; and one melting curve cycle. GAPDH was used as the internal control for qPCR. Relative gene expression was calculated using the 2^−^^ΔΔ^*^CT^* method. Feces samples were snap-frozen in liquid nitrogen and stored at −80 °C. DNA was extracted using a QIAamp DNA Stool Mini Kit (Qiagen, Hilden, Germany). Determination of *P. goldsteinii* abundance in fecal DNA was performed by real-time qPCR using *P. goldsteinii* 16S rRNA gene specific primers listed in Supplementary Table S2. The 16S rRNA gene V3−V4 primers were used as the internal control for qPCR assay.

### 4.7. RNA Sequencing and Bioinformatics Analyses

Total RNA was prepared from the intestine and hippocampus tissues of mice. A total of 11 colon (CTL, n = 3; MIAO, n = 4; MIAO + Pg, n = 4) and 10 hippocampus (CTL, n = 4; MIAO, n = 3; MIAO + Pg, n = 3) tissues were subjected for transcriptomic analysis. RNA sequencing was conducted on an Illumina HiSeq4000 using a paired-end run (2 × 150 bases). We used the STAR (v2.7.3a) two-pass mapping strategy to align the raw FASTQ reads against the mouse reference genome (GENCODE Mouse M24) downloaded from the GENCODE database. DESeq2 (v1.26) was used to normalize the raw read counts quantified by STAR with GENCODE Mouse M24 gene annotation. To perform gene set enrichment analysis (GSEA), we first converted gene expression data into the pre-ranked format by calculating the log2 fold change of different conditions. Next, we used GSEA software (v4.1.0) and MSigDB (v7.2) (http://www.gsea-msigdb.org/gsea/msigdb/) (accessed on March 2022) with the pre-rank mode to calculate the normalized enrichment scores and false-discovery rate (FDR). In particular, we used GO gene sets (MSigDB C5). Gene sets were considered significantly enriched at FDR < 0.25 when using Signal2Noise as a metric and 1000 permutations of gene sets. The dot plots displaying the most significantly upregulated (normalized enrichment score [*NES*] > 1) and downregulated (NES < −1) gene sets were generated using the ggplot function of the ggplot2 R package.

### 4.8. Statistical Analysis

Data are presented as the mean ± standard deviation. Because each group in the behavioral tests had at least 30 observations, data sets were assessed by parametric one-way ANOVA with Tukey’s post hoc multiple comparisons. The normality of other data in this study was confirmed by using the Shapiro–Wilk test. Differences between two groups were assessed using an unpaired two-tailed Student’s *t*-test. Data sets involving more than two groups were assessed by one-way ANOVA with Tukey’s post hoc multiple comparisons.

## Data Availability

The sequence reads of transcriptomic analysis were deposited under NCBI BioProject numbers PRJNA871204 and PRJNA871205. All relevant data is contained within the article. The original contributions presented in the study are included in the article/Supplementary Material, further inquiries can be directed to the corresponding authors.

## Supplementary Materials

The following supporting information can be downloaded at: https://www.mdpi.com/article/10.3390/ijms232113070/s1.

## References

[1] Matson J.L., Rieske R.D., Williams L.W. (2013). The relationship between autism spectrum disorders and attention-deficit/hyperactivity disorder: An overview. Res. Dev. Disabil..

[2] Kerns C.M., Kendall P.C., Berry L., Souders M.C., Franklin M.E., Schultz R.T., Miller J., Herrington J. (2014). Traditional and atypical presentations of anxiety in youth with autism spectrum disorder. J. Autism Dev. Disord..

[3] Maenner M.J., Shaw K.A., Bakian A.V., Bilder D.A., Durkin M.S., Esler A., Furnier S.M., Hallas L., Hall-Lande J., Hudson A. (2012). Prevalence of autism spectrum disorders—Autism and Developmental Disabilities Monitoring Network, 14 sites, United States, 2008. MMWR Surveill. Summ..

[4] Fernell E., Eriksson M.A., Gillberg C. (2013). Early diagnosis of autism and impact on prognosis: A narrative review. Clin. Epidemiol..

[5] DelaCuesta-Barrutia J., Penagarikano O., Erdozain A.M. (2020). G Protein-Coupled Receptor Heteromers as Putative Pharmacotherapeutic Targets in Autism. Front. Cell Neurosci..

[6] Theoharides T.C., Kempuraj D., Redwood L. (2009). Autism: An emerging ‘neuroimmune disorder’ in search of therapy. Expert. Opin. Pharmacother..

[7] Herbert M.R. (2010). Contributions of the environment and environmentally vulnerable physiology to autism spectrum disorders. Curr. Opin. Neurol..

[8] Ornoy A., Weinstein-Fudim L., Ergaz Z. (2016). Genetic Syndromes, Maternal Diseases and Antenatal Factors Associated with Autism Spectrum Disorders (ASD). Front. Neurosci..

[9] Parikshak N.N., Swarup V., Belgard T.G., Irimia M., Ramaswami G., Gandal M.J., Hartl C., Leppa V., Ubieta L.T., Huang J. (2016). Genome-wide changes in lncRNA, splicing, and regional gene expression patterns in autism. Nature.

[10] Prosperi M., Turi M., Guerrera S., Napoli E., Tancredi R., Igliozzi R., Apicella F., Valeri G., Lattarulo C., Gemma A. (2020). Sex Differences in Autism Spectrum Disorder: An Investigation on Core Symptoms and Psychiatric Comorbidity in Preschoolers. Front. Integr. Neurosci..

[11] Boksa P. (2010). Effects of prenatal infection on brain development and behavior: A review of findings from animal models. Brain Behav. Immun..

[12] Sotgiu S., Manca S., Gagliano A., Minutolo A., Melis M.C., Pisuttu G., Scoppola C., Bolognesi E., Clerici M., Guerini F.R. (2020). Immune regulation of neurodevelopment at the mother-foetus interface: The case of autism. Clin. Transl. Immunol..

[13] Han V.X., Patel S., Jones H.F., Nielsen T.C., Mohammad S.S., Hofer M.J., Gold W., Brilot F., Lain S.J., Nassar N. (2021). Maternal acute and chronic inflammation in pregnancy is associated with common neurodevelopmental disorders: A systematic review. Transl. Psychiatry.

[14] Depino A.M. (2015). Early prenatal exposure to LPS results in anxiety- and depression-related behaviors in adulthood. Neuroscience.

[15] Aria F., Bonini S.A., Cattaneo V., Premoli M., Mastinu A., Maccarinelli G., Memo M. (2020). Brain Structural and Functional Alterations in Mice Prenatally Exposed to LPS Are Only Partially Rescued by Anti-Inflammatory Treatment. Brain Sci..

[16] Bergdolt L., Dunaevsky A. (2019). Brain changes in a maternal immune activation model of neurodevelopmental brain disorders. Prog. Neurobiol..

[17] Kalish B.T., Kim E., Finander B., Duffy E.E., Kim H., Gilman C.K., Yim Y.S., Tong L., Kaufman R.J., Griffith E.C. (2021). Maternal immune activation in mice disrupts proteostasis in the fetal brain. Nat. Neurosci..

[18] Li Q., Han Y., Dy A.B.C., Hagerman R.J. (2017). The Gut Microbiota and Autism Spectrum Disorders. Front. Cell Neurosci..

[19] Foster J.A., McVey Neufeld K.A. (2013). Gut-brain axis: How the microbiome influences anxiety and depression. Trends Neurosci..

[20] Eisenstein M. (2016). Microbiome: Bacterial broadband. Nature.

[21] Abdellatif B., McVeigh C., Bendriss G., Chaari A. (2020). The Promising Role of Probiotics in Managing the Altered Gut in Autism Spectrum Disorders. Int. J. Mol. Sci..

[22] Sgritta M., Dooling S.W., Buffington S.A., Momin E.N., Francis M.B., Britton R.A., Costa-Mattioli M. (2019). Mechanisms Underlying Microbial-Mediated Changes in Social Behavior in Mouse Models of Autism Spectrum Disorder. Neuron.

[23] Kaluzna-Czaplinska J., Blaszczyk S. (2012). The level of arabinitol in autistic children after probiotic therapy. Nutrition.

[24] Santocchi E., Guiducci L., Prosperi M., Calderoni S., Gaggini M., Apicella F., Tancredi R., Billeci L., Mastromarino P., Grossi E. (2020). Effects of Probiotic Supplementation on Gastrointestinal, Sensory and Core Symptoms in Autism Spectrum Disorders: A Randomized Controlled Trial. Front. Psychiatry.

[25] O’Toole P.W., Marchesi J.R., Hill C. (2017). Next-generation probiotics: The spectrum from probiotics to live biotherapeutics. Nat. Microbiol..

[26] Chang C.J., Lin T.L., Tsai Y.L., Wu T.R., Lai W.F., Lu C.C., Lai H.C. (2019). Next generation probiotics in disease amelioration. J. Food Drug Anal..

[27] Hsiao E.Y., McBride S.W., Hsien S., Sharon G., Hyde E.R., McCue T., Codelli J.A., Chow J., Reisman S.E., Petrosino J.F. (2013). Microbiota modulate behavioral and physiological abnormalities associated with neurodevelopmental disorders. Cell.

[28] Xu M., Xu X., Li J., Li F. (2019). Association Between Gut Microbiota and Autism Spectrum Disorder: A Systematic Review and Meta-Analysis. Front. Psychiatry.

[29] Sharon G., Cruz N.J., Kang D.W., Gandal M.J., Wang B., Kim Y.M., Zink E.M., Casey C.P., Taylor B.C., Lane C.J. (2019). Human Gut Microbiota from Autism Spectrum Disorder Promote Behavioral Symptoms in Mice. Cell.

[30] Chang C.J., Lin C.S., Lu C.C., Martel J., Ko Y.F., Ojcius D.M., Tseng S.F., Wu T.R., Chen Y.Y., Young J.D. (2015). Ganoderma lucidum reduces obesity in mice by modulating the composition of the gut microbiota. Nat. Commun..

[31] Wu T.R., Lin C.S., Chang C.J., Lin T.L., Martel J., Ko Y.F., Ojcius D.M., Lu C.C., Young J.D., Lai H.C. (2019). Gut commensal *Parabacteroides goldsteinii* plays a predominant role in the anti-obesity effects of polysaccharides isolated from Hirsutella sinensis. Gut.

[32] Lai H.C., Lin T.L., Chen T.W., Kuo Y.L., Chang C.J., Wu T.R., Shu C.C., Tsai Y.H., Swift S., Lu C.C. (2022). Gut microbiota modulates COPD pathogenesis: Role of anti-inflammatory *Parabacteroides goldsteinii* lipopolysaccharide. Gut.

[33] Prut L., Belzung C. (2003). The open field as a paradigm to measure the effects of drugs on anxiety-like behaviors: A review. Eur. J. Pharmacol..

[34] Bourin M., Hascoet M. (2003). The mouse light/dark box test. Eur. J. Pharmacol..

[35] Pellow S., Chopin P., File S.E., Briley M. (1985). Validation of open:closed arm entries in an elevated plus-maze as a measure of anxiety in the rat. J. Neurosci. Methods.

[36] Kim D.G., Gonzales E.L., Kim S., Kim Y., Adil K.J., Jeon S.J., Cho K.S., Kwon K.J., Shin C.Y. (2019). Social Interaction Test in Home Cage as a Novel and Ethological Measure of Social Behavior in Mice. Exp. Neurobiol..

[37] Yang M., Silverman J.L., Crawley J.N. (2011). Automated three-chambered social approach task for mice. Curr. Protoc. Neurosci..

[38] Li W., Chen M., Feng X., Song M., Shao M., Yang Y., Zhang L., Liu Q., Lv L., Su X. (2021). Maternal immune activation alters adult behavior, intestinal integrity, gut microbiota and the gut inflammation. Brain Behav..

[39] Choi G.B., Yim Y.S., Wong H., Kim S., Kim H., Kim S.V., Hoeffer C.A., Littman D.R., Huh J.R. (2016). The maternal interleukin-17a pathway in mice promotes autism-like phenotypes in offspring. Science.

[40] Fattorusso A., Di Genova L., Dell’Isola G.B., Mencaroni E., Esposito S. (2019). Autism Spectrum Disorders and the Gut Microbiota. Nutrients.

[41] Muller P.A., Schneeberger M., Matheis F., Wang P., Kerner Z., Ilanges A., Pellegrino K., Del Marmol J., Castro T.B.R., Furuichi M. (2020). Microbiota modulate sympathetic neurons via a gut-brain circuit. Nature.

[42] Scott G.A., Terstege D.J., Vu A.P., Law S., Evans A., Epp J.R. (2020). Disrupted Neurogenesis in Germ-Free Mice: Effects of Age and Sex. Front Cell Dev. Biol..

[43] Chaddad A., Desrosiers C., Hassan L., Tanougast C. (2017). Hippocampus and amygdala radiomic biomarkers for the study of autism spectrum disorder. BMC Neurosci..

[44] Wasilewska J., Klukowski M. (2015). Gastrointestinal symptoms and autism spectrum disorder: Links and risks—a possible new overlap syndrome. Pediatric. Health Med. Ther..

[45] Dinan T.G., Cryan J.F. (2017). Gut instincts: Microbiota as a key regulator of brain development, ageing and neurodegeneration. J. Physiol..

[46] Kelly J.R., Minuto C., Cryan J.F., Clarke G., Dinan T.G. (2017). Cross Talk: The Microbiota and Neurodevelopmental Disorders. Front. Neurosci..

[47] Ma Q., Xing C., Long W., Wang H.Y., Liu Q., Wang R.F. (2019). Impact of microbiota on central nervous system and neurological diseases: The gut-brain axis. J. Neuroinflammation.

[48] Critchfield J.W., van Hemert S., Ash M., Mulder L., Ashwood P. (2011). The potential role of probiotics in the management of childhood autism spectrum disorders. Gastroenterol. Res. Pract..

[49] Lin T.L., Shu C.C., Lai W.F., Tzeng C.M., Lai H.C., Lu C.C. (2019). Investiture of next generation probiotics on amelioration of diseases—Strains do matter. Med. Microecol..

[50] Tsai Y.L., Lin T.L., Chang C.J., Wu T.R., Lai W.F., Lu C.C., Lai H.C. (2019). Probiotics, prebiotics and amelioration of diseases. J. Biomed. Sci..

[51] Liu Y.W., Liong M.T., Chung Y.E., Huang H.Y., Peng W.S., Cheng Y.F., Lin Y.S., Wu Y.Y., Tsai Y.C. (2019). Effects of Lactobacillus plantarum PS128 on Children with Autism Spectrum Disorder in Taiwan: A Randomized, Double-Blind, Placebo-Controlled Trial. Nutrients.

[52] Ait-Belgnaoui A., Colom A., Braniste V., Ramalho L., Marrot A., Cartier C., Houdeau E., Theodorou V., Tompkins T. (2014). Probiotic gut effect prevents the chronic psychological stress-induced brain activity abnormality in mice. Neurogastroenterol. Motil..

[53] Bravo J.A., Forsythe P., Chew M.V., Escaravage E., Savignac H.M., Dinan T.G., Bienenstock J., Cryan J.F. (2011). Ingestion of Lactobacillus strain regulates emotional behavior and central GABA receptor expression in a mouse via the vagus nerve. Proc. Natl. Acad. Sci. USA.

[54] Chao L., Liu C., Sutthawongwadee S., Li Y., Lv W., Chen W., Yu L., Zhou J., Guo A., Li Z. (2020). Effects of Probiotics on Depressive or Anxiety Variables in Healthy Participants Under Stress Conditions or With a Depressive or Anxiety Diagnosis: A Meta-Analysis of Randomized Controlled Trials. Front. Neurol..

[55] Cheng L.H., Liu Y.W., Wu C.C., Wang S., Tsai Y.C. (2019). Psychobiotics in mental health, neurodegenerative and neurodevelopmental disorders. J. Food Drug Anal..

[56] Chan J.L., Wu S., Geis A.L., Chan G.V., Gomes T.A.M., Beck S.E., Wu X., Fan H., Tam A.J., Chung L. (2019). Non-toxigenic *Bacteroides fragilis* (NTBF) administration reduces bacteria-driven chronic colitis and tumor development independent of polysaccharide A. Mucosal Immunol..

[57] Zhang W., Zhu B., Xu J., Liu Y., Qiu E., Li Z., Li Z., He Y., Zhou H., Bai Y. (2018). *Bacteroides fragilis* Protects Against Antibiotic-Associated Diarrhea in Rats by Modulating Intestinal Defenses. Front Immunol..

[58] Sun F., Zhang Q., Zhao J., Zhang H., Zhai Q., Chen W. (2019). A potential species of next-generation probiotics? The dark and light sides of *Bacteroides fragilis* in health. Food Res. Int..

[59] Jyonouchi H., Geng L., Ruby A., Zimmerman-Bier B. (2005). Dysregulated innate immune responses in young children with autism spectrum disorders: Their relationship to gastrointestinal symptoms and dietary intervention. Neuropsychobiology.

[60] Lee M., Krishnamurthy J., Susi A., Sullivan C., Gorman G.H., Hisle-Gorman E., Erdie-Lalena C.R., Nylund C.M. (2018). Association of Autism Spectrum Disorders and Inflammatory Bowel Disease. J. Autism Dev. Disord..

[61] Bertolino B., Crupi R., Impellizzeri D., Bruschetta G., Cordaro M., Siracusa R., Esposito E., Cuzzocrea S. (2017). Beneficial Effects of Co-Ultramicronized Palmitoylethanolamide/Luteolin in a Mouse Model of Autism and in a Case Report of Autism. CNS Neurosci. Ther..

[62] Serra D., Henriques J.F., Sousa F.J., Laranjo M., Resende R., Ferreira-Marques M., de Freitas V., Silva G., Peca J., Dinis T.C.P. (2022). Attenuation of Autism-like Behaviors by an Anthocyanin-Rich Extract from Portuguese Blueberries via Microbiota-Gut-Brain Axis Modulation in a Valproic Acid Mouse Model. Int. J. Mol. Sci..

[63] Thye M.D., Bednarz H.M., Herringshaw A.J., Sartin E.B., Kana R.K. (2018). The impact of atypical sensory processing on social impairments in autism spectrum disorder. Dev. Cogn. Neurosci..

[64] Padgett C.L., Slesinger P.A. (2010). GABAB receptor coupling to G-proteins and ion channels. Adv. Pharmacol..

[65] Wicker B., Monfardini E., Royet J.P. (2016). Olfactory processing in adults with autism spectrum disorders. Mol. Autism.

[66] Ebert D.H., Greenberg M.E. (2013). Activity-dependent neuronal signalling and autism spectrum disorder. Nature.

[67] Rojas D.C. (2014). The role of glutamate and its receptors in autism and the use of glutamate receptor antagonists in treatment. J. Neural Transm..

[68] Foss-Feig J.H., Adkinson B.D., Ji J.L., Yang G., Srihari V.H., McPartland J.C., Krystal J.H., Murray J.D., Anticevic A. (2017). Searching for Cross-Diagnostic Convergence: Neural Mechanisms Governing Excitation and Inhibition Balance in Schizophrenia and Autism Spectrum Disorders. Biol. Psychiatry.

[69] Fernandez M., Mollinedo-Gajate I., Penagarikano O. (2018). Neural Circuits for Social Cognition: Implications for Autism. Neuroscience.

[70] Torrente F., Ashwood P., Day R., Machado N., Furlano R.I., Anthony A., Davies S.E., Wakefield A.J., Thomson M.A., Walker-Smith J.A. (2002). Small intestinal enteropathy with epithelial IgG and complement deposition in children with regressive autism. Mol. Psychiatry.

[71] Wang M., Wei P.C., Lim C.K., Gallina I.S., Marshall S., Marchetto M.C., Alt F.W., Gage F.H. (2020). Increased Neural Progenitor Proliferation in a hiPSC Model of Autism Induces Replication Stress-Associated Genome Instability. Cell Stem Cell.

[72] Huang J.Y., Tian Y., Wang H.J., Shen H., Wang H., Long S., Liao M.H., Liu Z.R., Wang Z.M., Li D. (2016). Functional Genomic Analyses Identify Pathways Dysregulated in Animal Model of Autism. CNS Neurosci. Ther..

[73] Cheng P., Qiu Z., Du Y. (2021). Potassium channels and autism spectrum disorder: An overview. Int. J. Dev. Neurosci..

[74] Hetman M., Slomnicki L.P. (2019). Ribosomal biogenesis as an emerging target of neurodevelopmental pathologies. J. Neurochem..

[75] Varga N.A., Pentelenyi K., Balicza P., Gezsi A., Remenyi V., Harsfalvi V., Bencsik R., Illes A., Prekop C., Molnar M.J. (2018). Mitochondrial dysfunction and autism: Comprehensive genetic analyses of children with autism and mtDNA deletion. Behav. Brain Funct..

[76] Balachandar V., Rajagopalan K., Jayaramayya K., Jeevanandam M., Iyer M. (2021). Mitochondrial dysfunction: A hidden trigger of autism?. Genes Dis..

[77] Hsueh P.T., Lin H.H., Wang H.H., Liu C.L., Ni W.F., Liu J.K., Chang H.H., Sun D.S., Chen Y.S., Chen Y.L. (2018). Immune imbalance of global gene expression, and cytokine, chemokine and selectin levels in the brains of offspring with social deficits via maternal immune activation. Genes Brain Behav..

