# Supplementary material for: Amelioration of Maternal Immune Activation-Induced Autism Relevant Behaviors by Gut Commensal Parabacteroides goldsteinii
Source: Int J Mol Sci. 2022 Oct 28;23(21):13070. doi: 10.3390/ijms232113070 (PMC9657948; doi:10.3390/ijms232113070)
Supplement: Supplementary file 1 [file ijms-23-13070-s001.zip › Supplementary materials_20221025_clean.pdf]

Supplementary Figure S1. The abundance of *P. goldsteinii* in the feces of MIAO and MIAO+Pg groups.

The relative abundance of *P. goldsteinii* (relative to total 16S rRNA) in the feces of MIAO and MIAO+Pg groups was determined by using qPCR. Data were presented as the mean ± standard deviation. *, $p < 0.05$ (unpaired Student's t-test). MIAO+Pg, MIAO treated with *P. goldsteinii* MTS01.

Supplementary Figure S2. Transcriptome alterations in the colon of lipopolysaccharide-induced MIAO.

Dot plots of the significantly upregulated (normalized enrichment score [NES] > 1 and false-discovery rate [FDR] q value < 0.25) and downregulated ([NES] < −1 and FDR q value < 0.25) gene sets from gene set enrichment analysis of the MIAO group compared to the CTL (top 20 gene sets in GO_BP, GO_MF and GO_CC) are presented in A and B, respectively. Gene sets with similar functions were labeled in color, and the functions were also described.

Supplementary Figure S3. Transcriptome alterations in hippocampus of lipopolysaccharide-induced MIAO.

Dot plots of the significantly upregulated (normalized enrichment score [NES] > 1 and false-discovery rate [FDR] q value < 0.25) and downregulated ([NES] < −1 and FDR q value < 0.25) gene sets from gene set enrichment analysis of the MIAO group compared to the CTL (top 20 gene sets in GO_BP, GO_MF and GO_CC) are presented in A and B, respectively. Gene sets with similar functions were labeled in color, and the functions were also described.

Supplementary Figure S4. Transcriptome alterations in the colon of germ-free mice following colonization with *P. goldsteinii* MTS01.

Dot plots of the top 20 significantly altered pathways (GO_BP) in the colon of germ-free mice

with *P. goldsteinii* MTS01 from gene set enrichment analysis are presented. The upregulated (normalized enrichment score [NES] > 1) and downregulated (NES < −1) gene sets in contrast to control mice are both presented.

Supplementary Figure S5. Hypothetical model of the ameliorative effects of *P. goldsteinii* MTS01 (Pg MTS01) in MIAO.

Pregnant mice were injected with lipopolysaccharide to induce maternal immune activation (MIA). Mice offspring were either untreated (MIAO, left panel) or orally gavaged with live Pg MTS01 (MIAO+Pg, right panel). Anxiety-like behaviors and social behavioral deficits in MIAO mice were significantly ameliorated by the administration of Pg MTS01 (red block). Transcriptomic analysis in the colon (blue block) and hippocampus (green block) revealed multiple modes of action of Pg MTS01.

Supplementary Table S1. Design and assignment of mice experiments.

Supplementary Table S2. Primers used in this study.

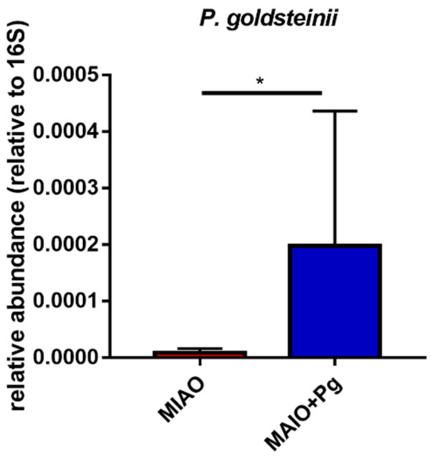

Supplementary Figure S1

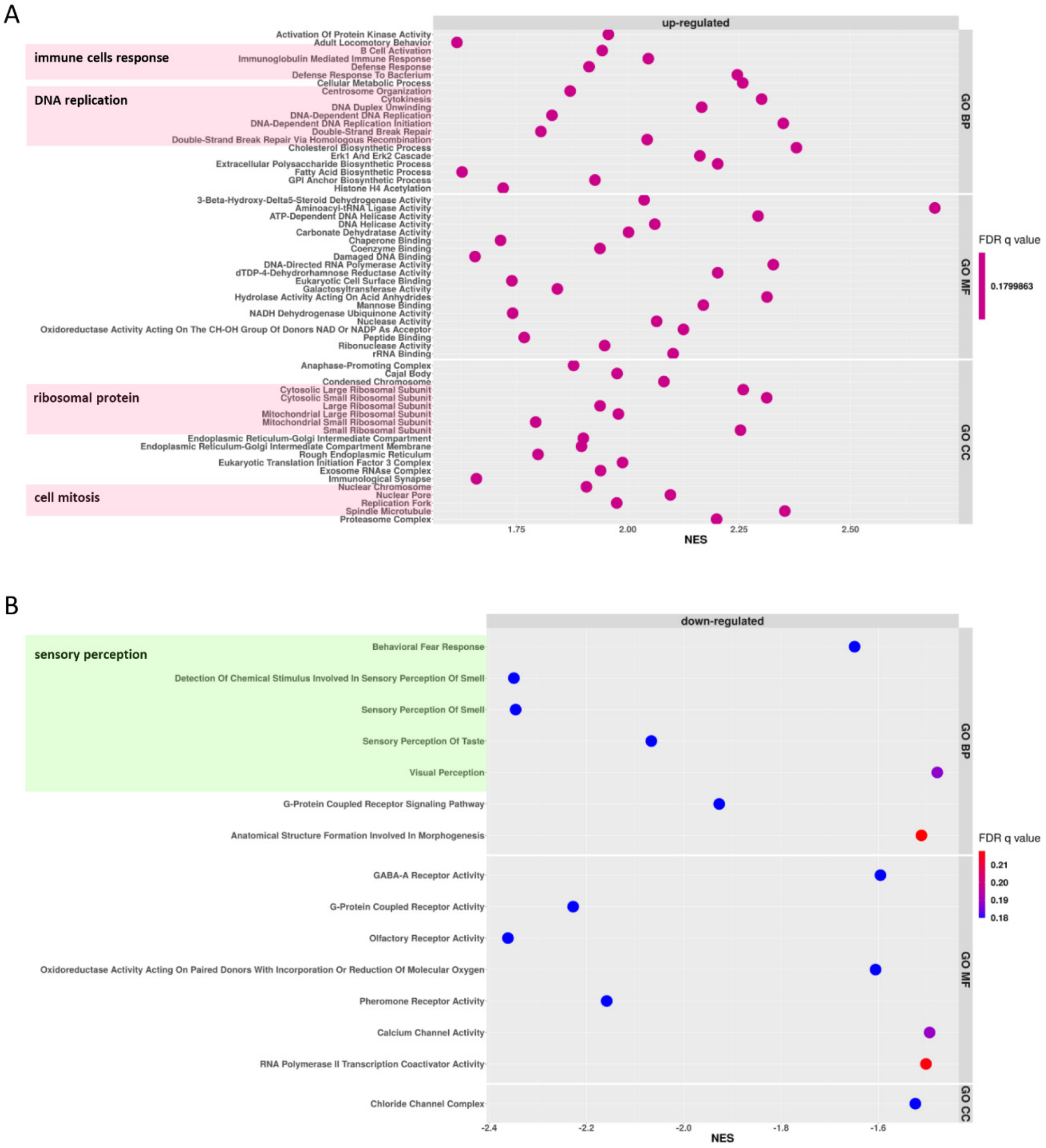

Supplementary Figure S2

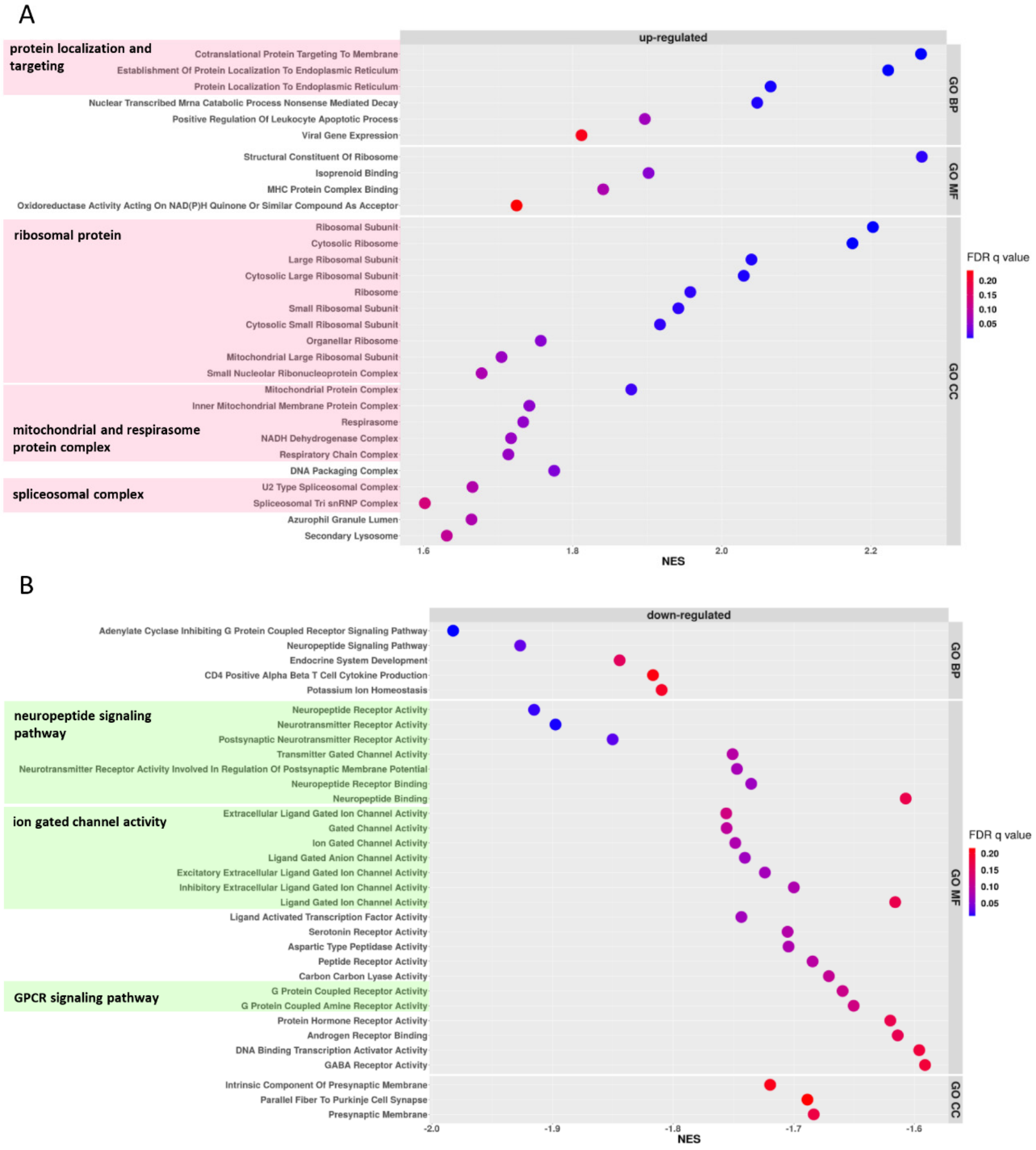

Supplementary Figure S3

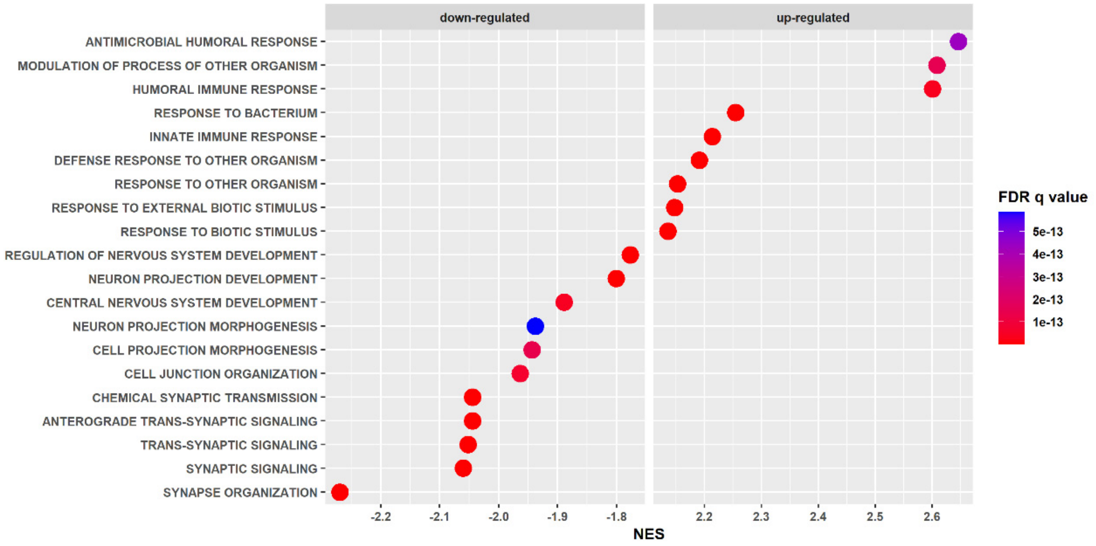

Supplementary Figure S4

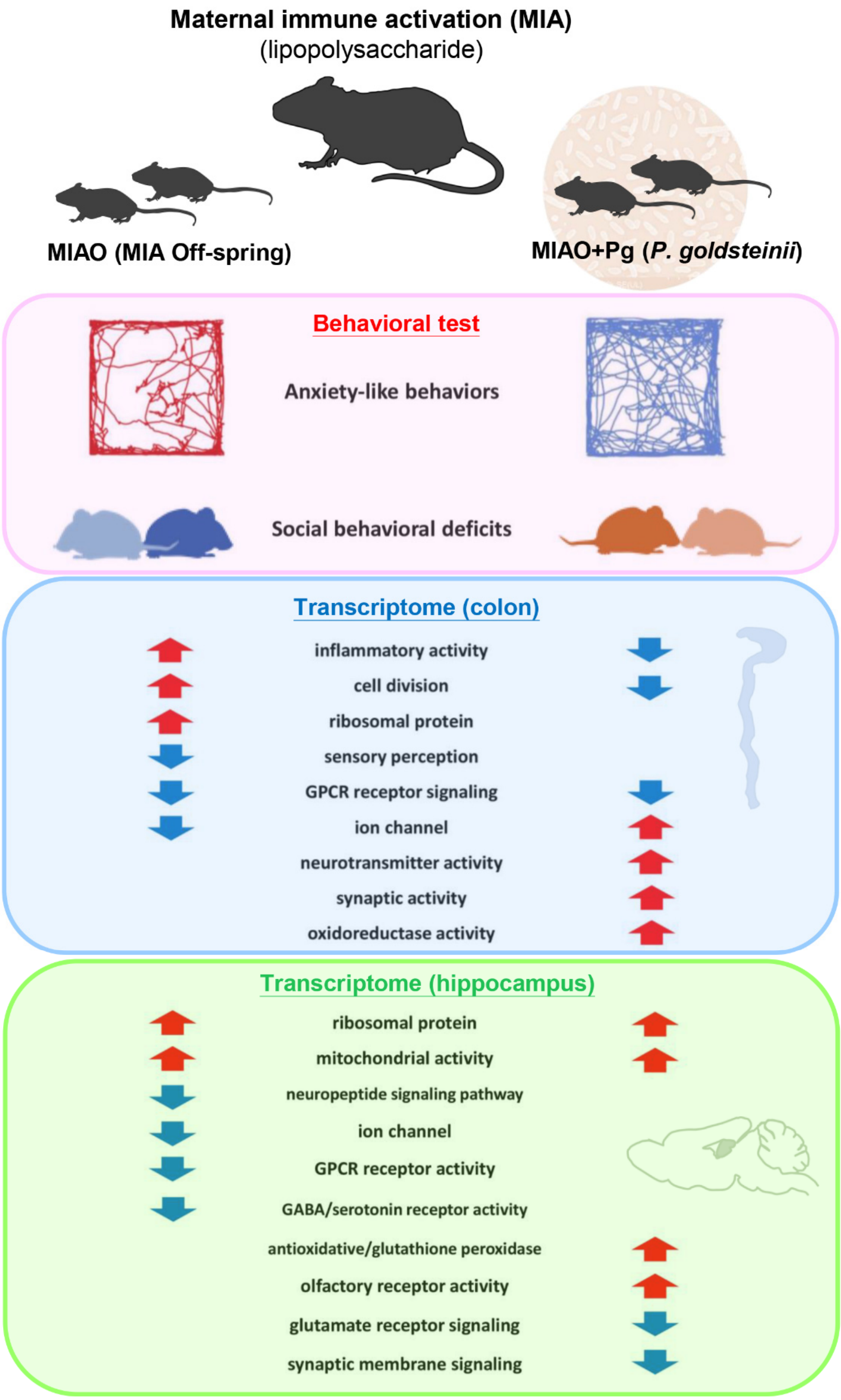

Supplementary Figure S5

Supplementary Table S2. Primers used in this study.

Target	Primer sequence (5'–3')	
IL-1β	Forward	TTGAAGAAGAGCCCATCCTC
	Reverse	CAGCTCATATGGGTCCGAC
TNF-α	Forward	TAGCCAGGAGGGAGAACAGA
	Reverse	TTTTCTGGAGGGAGATGTGG
IL-6	Forward	CCGGAGAGGAGACTTCAC
	Reverse	TCCACGATTTCCCAGAGA
Rpl34	Forward	GCACCTAAATCTGCATGTGGCG
	Reverse	TGTCACGGACACACTTGGCACA
Rpl11	Forward	GAGAGCGGAGACAGACTGACC
	Reverse	GGATGCCAAAGGACCTGACAGT
Wdr93	Forward	TCGTCTGTGAGGATGGTGTGCT
	Reverse	TGCACCAGGAAGAATCGGAGGA
Ndufa3	Forward	TGCCCATGATTAGCCCCTACAC
	Reverse	TGTTCCCGTCATCTCTCACAGG
Duox2	Forward	GAGAAAGGCTGTGACCAAGCAG
	Reverse	TCACGCACTTGCTGGGATGAGT
Avpr1A	Forward	CATCCTCTGCTGGACACCTTTC
	Reverse	TCAAGGAAGCCAGTAACGCCGT
Crh	Forward	GGAATCTCAACAGAAGTCCCGC
	Reverse	CTGCAGCAACACGCGGAAAAAG
Grm6	Forward	GCAACCAATGGAAGTGCCAGCA
	Reverse	TCGCCTGACCACTGTAGAGCTT
CD79a	Forward	ACGCTCCTGTGGTACTTACCTC
	Reverse	CCTTCTGCTGTGATGATGCGGT
CD79b	Forward	CCAGCAATGACAAGCAGTGACC
	Reverse	CCTGAGTGGTTTGTGTAGCAGTG
TLR4	Forward	GAGCCGG AAGGTTA TTGTGGT
	Reverse	CCTCTGC TGTTTGC TCAGGAT
Ccl8	Forward	GGGTGCTGAAAAGCTACGAGAG
	Reverse	GGATCTCCATGTACTCACTGACC
Rims2	Forward	GATGTCTCCTCATTGCCACTCC
	Reverse	ATCCTCGCAGTCGTAGTCAGAC
GAPDH	Forward	GCATCCACTGGTGCTGCC
	Reverse	TCATCATACTTGGCAGGTTTC
16S V3–V4	Forward	CCTACGGGNGGCWGCAG
	Reverse	GACTACHVGGGTATCTAATCC
PG 16S	Forward	GAATAAAGTGAGGAACGTGTT
	Reverse	AACTTTCACCGCTGACTTAATTA
